# Interference Suppression Algorithm Based on Short Time Fractional Fourier Transform

**DOI:** 10.3390/s24061785

**Published:** 2024-03-10

**Authors:** Xiaolu Guo, Jia Su, Nan Zhu

**Affiliations:** 1 School of Electronic Information Engineering, Xi’an Technological University, Xi’an 710021, China; 2 School of Electronics and Information, Northwestern Polytechnical University, Xi’an 710072, China

**Keywords:** interference suppression, short time, fractional Fourier transform

## Abstract

Narrowband interference and wideband interference are both common jamming signals against synthetic aperture radar, which can degrade the signal severely. To suppress interference effectively, an interference suppression method based on short-time fractional Fourier transform (STFrFT) is proposed. After transforming the signal into the time–frequency domain through STFrFT, an adaptive gain coefficient is determined for the instantaneous frequency spectrum at every certain time. The gain coefficient can be preserved while suppressing the interference. Finally, we obtain the useful signal by inverse STFrFT. The simulation and performance analysis show the effectiveness and validity of the proposed algorithm for measured data.

## 1. Introduction

Radar is an electromagnetic sensor used for detecting, locating, tracking, and recognizing objects of various kinds at considerable distances. In civilian and military applications, synthetic aperture radar (SAR) is remarkable for its high resolution and wide surveillance swath [1,2,3,4,5,6,7,8]. However, in complex electromagnetic interference environments, the ability of radar systems to obtain information will be significantly reduced. To further improve the detection performance of targets, it is necessary to perform interference suppression preprocessing before detecting the raw echo data.

Usually, unintentional interferences are classified into narrowband interference (NBI) and wideband interference (WBI) based on the bandwidth of the interference. NBI is interference with a bandwidth of less than 1% of the signal bandwidth [9,10]. The bandwidth of the interference is relatively smaller (usually less than 1%) than that of the target signal. On the contrary, WBI is interference with a bandwidth greater than 1%.

NBI suppression methods can be divided into two categories. The first type involves adaptive filtering method [11,12,13] and the frequency notch filtering method [14,15,16,17,18,19]. This algorithm transforms the signal into the frequency domain to perform interference notch filtering. This type of method is simple to run but has drawbacks: Firstly, if the signal and interference are relatively close or overlap in the spectrum, the energy of the target signal will also be weakened when nulling. Secondly, it leads to high sidelobes, resulting in false alarms and false targets. This method searches for the interference parameters in the time domain and reconstructs the interference signal using the search results. Interference suppression will be achieved by canceling the reconstructed signal from the original echo signal. The second type of NBI suppression method involves the subspace projection method [9,20,21,22] and parameterized interference suppression method [14,23]. This type of method searches for the interference parameters in the time domain and reconstructs the interference signal using the search results. Interference suppression will be achieved by canceling the reconstructed signal from the original echo signal.

WBI is interference with a bandwidth greater than 1% of the signal bandwidth. WBI highly overlaps with useful signals in both the time and frequency domains, making it difficult to effectively suppress interference through one-dimensional time or frequency domain methods. Therefore, the idea of WBI suppression methods differs from NBI suppression and can be divided into two categories. The first type utilizes mathematical theory to transform signals into the spatial or time–frequency domains, where interference exhibits good time–frequency clustering. Using this advantage, filters are designed to suppress interference signals. Such mathematical theories include Wigner Distribution [24,25], fractional Fourier transform (FrFT) [26,27,28,29,30], short-time Fourier transform (STFT) [31,32,33], or wavelets [31,34,35]. The second type of method models interference as high-order polynomials, reconstrues it, and then cancels it from the original interference. The parameters required for modeling are estimated based on different criteria [36].

In response to the above issues, an interference suppression algorithm based on short-time fractional Fourier transform is proposed. Firstly, we approximate non-stationary signals and stationary signals using short-time theory. Secondly, the excellent time–frequency (TF) clustering of FrFT on the TF plane is used to distinguish interference and signals. Finally, we design a gain control coefficient to protect the signal and suppress interference. The method presented in this article can suppress linear frequency modulation interference in traditional NBI and WBI in unintentional interference.

## 2. Model and Theory

### 2.1. Signal Model

When the signal bandwidth is in the same frequency band as NBI and WBI, the detection performance of the radar system will be significantly reduced, thereby increasing the difficulty of post-processing. The SAR echo data with interference can be modeled as

(1) x(t)=S(t)+I(t)+N(t)

where *S*(*t*), *I*(*t*), and *N*(*t*) are the useful target signal, interference, and Gaussian white noise, respectively; *t* represents fast time. For NBI, its interference model can be formulated as [37]

(2) $$I_{NBI}(t)=\sum_{l=1}^{L}a_{l}(t)exp\left\{j\left(2\pi f_{l}t+\varphi_{l}\right)\right\}$$

where *a_l_*(*t*), *f_l_*, and *φ_l_* are the *l*-th amplitude, frequency, and phase, respectively, and *L* is the number of NBIs. With the continuous increase in electronic devices, radar systems are facing more and more WBI, among which linear frequency modulation (LFM) interference is one of the main types of WBI. Other complex interference signals with high-order phases can be processed in segments to approximate LFM characteristics during the time period. The signal model [19] can be denoted as

(3) $$I_{WBI}\left(t\right)=\sum_{l=1}^{L}a_{l}\left(t\right)\;exp\left\{j(2\pi f_{l}t+\pi \gamma_{1}t^{2}+\varphi_{l})\right\}$$

where *γ_l_* represents the chirp rate of the *l*-th WBI interference.

### 2.2. Short-Time Fractional Fourier Transform

A commonly used method for non-stationary signals is short-time Fourier transform. Assuming that the non-stationary signal *x*(*t*) is approximately considered stationary within the window function *h*(*t*), the traditional method of stationary signals can be used for analysis in a short period of time. However, signals may still be non-stationary in a short time in practice. Therefore, by combining short time and FRFT, a short-term fractional Fourier transform is obtained, which is defined as follows:

(4) $$STFrFT_{p}(t,f)=\int_{-\infty }^{\infty }x\left(\tau \right)\;h^{*}\left(t-\tau \right)K_{p}(\tau ,u)d\tau$$

where

(5) $$K_{p}(\tau ,u)=\left\{\begin{array}{c} \sqrt{\left(1-jcot\alpha \right)}e^{j\pi \left(\tau^{2}cot\alpha -2u\tau csc\alpha +u^{2}cot\alpha \right)},\alpha \neq n\pi \\ \delta (\tau -u),\alpha =2n\pi \\ \delta (\tau +u),\alpha =\left(2n\pm 1\right)\pi \end{array}\right.$$

where *h*(·) is the window function, *p* ∈ (−2, 2] is the order of FrFT, and *α* = *pπ*/2 is the rotation angle. Taking WBI as an example, substituting Equation (3) into Equation (4) can obtain the short-term fractional Fourier transform expression of WBI:

(6) $$\begin{array}{l} STFrFT_{p}(t,f)=\int_{-\infty }^{\infty }I_{\mathrm{WBI}}\left(\tau \right)\;h^{*}\left(t-\tau \right)K_{p}(\tau ,u)d\tau \\ =\int_{-\infty }^{\infty }a\left(t\right)\;e^{j\varphi }e^{j\pi \tau^{2}(\gamma +cot\alpha )}e^{j2\pi \tau (f-ucsc\alpha )}h^{*}\left(t-\tau \right)d\tau \end{array}$$

The above formula indicates that when $\gamma_{l}=-\cot\alpha_{0}$ (i.e., $\alpha_{0}=-\mathrm{arccot}\gamma$), the WBI interference signal reaches its maximum value within a certain time window, and the WBI signal can be regarded as an NBI signal. Therefore, existing NBI suppression methods can be used to effectively identify and suppress WBI in a short time window. After interference suppression, it is necessary to use the short-term fractional Fourier inverse transform to obtain the echo data. Based on the reversibility of the fractional Fourier transform, the inverse transform of the short-term fractional Fourier transform can be expressed as

(7) $$S(t)={\left(STFrFT_{p}(t,f)\right)}^{-1}=STFrFT_{-p}(t,f)$$

where ${\left(\;\cdot \;\right)}^{-1}$ represents the inverse transformation. The above equation indicates that the invertibility of the short-term fractional Fourier transform can be achieved by transforming kernel functions.

## 3. Suppression Based on Short-Time Fractional Fourier Transform

**Figure 1:**
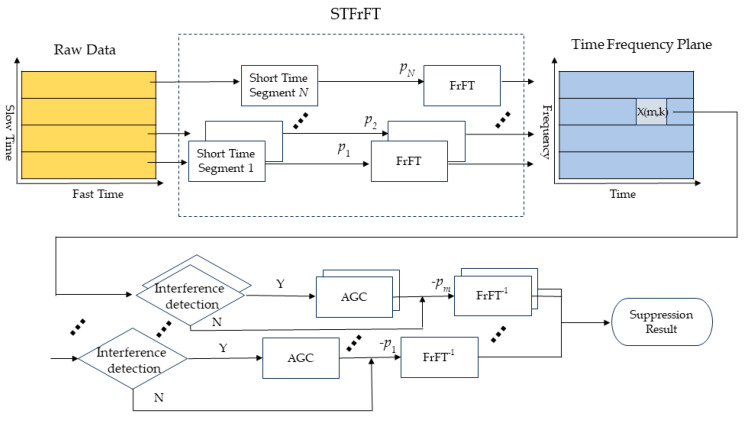
Flowchart of proposed algorithm.

The flowchart of the proposed algorithm is shown in Figure 1. In this flowchart, we first perform STFrFT on the fast time samples for each pulse (slow time) extracted from the raw data. Then, we perform interference detection for each datum *X*(*m*, *k*). If interference exists, adaptive gain control (AGC) is adopted to suppress the interference. Finally, after suppression, the inverse fractional Fourier transform is utilized to obtain the interference-free signal.

### 3.1. STFrFT Flow

Short-time fractional Fourier transform can effectively accumulate energy for LFM (or signals that approximate linear frequency modulation characteristics within a short time window). At the same time, adaptive gain control can suppress strong signal components while preserving the time–frequency characteristics of useful signals as much as possible. Therefore, an interference suppression algorithm based on short-term fractional Fourier transform is proposed, and the specific steps of the algorithm are as follows:

Step 1: Divide the echo signal into uniformly spaced, short time segments. In the first segment, search for the optimal order of the signal as *p*_1_, and perform *p*_1_-order FrFT on this segment. Because the signal is slow time-varying, the optimal order of the second segment, *p*_2_, can be searched for in the interval $\left[p_{1}-\Delta p,p_{1}+\Delta p\right]$. Perform FrFT on the optimal order *p*_2_ found in the search. By analogy, the optimal orders of each time segment, $p\in \left[p_{1},p_{2},\ldots ,p_{N}\right]$, is obtained. Subsequently, the short-time fractional Fourier transform of the echo signal, STFrFT_p_(*t*, *f*), is obtained.

Step 2: In the STFrFT domain, NBI and WBI have good time–frequency aggregation characteristics. Each time segment is detected sequentially. The NBI detection method proposed in reference [9] is used to detect interference in each segment. If there is an interference signal, an adaptive gain control (AGC) method is adopted to suppress the interference.

Step 3: The optimal order (*p*) obtained in step 1 is used to perform *p*-order FrFT (short-time fractional-order inverse transformation) on the data in each segment to obtain useful signals after interference suppression.

### 3.2. AGC

AGC can suppress the interference while maintaining the useful signal simultaneously by constructing a coefficient, W. As analyzed previously, whether it is NBI or WBI, it can be considered a narrowband signal in every time slice of the short fractional Fourier transform domain. The frequency notch method can be used to suppress interference signals, but the notch filtering method will lose useful data in the same frequency band as the interference signal while suppressing interference. Therefore, we strive to maintain the time–frequency characteristics of useful signals while suppressing interference by designing appropriate gain coefficients. Assume that the gain coefficient at the *k*-th frequency point in the *m*-th time slice in the short-time fractional Fourier transform domain is

(8) $$W(m,k)=\left\{\begin{array}{c} mid(X(m,:))/\left|X(m,k)\right|\\ 1 \end{array}\right.\;\begin{array}{c} ,\mathrm{with}\;\mathrm{interference}\\ ,\mathrm{without}\;\mathrm{interference} \end{array}$$

where *X*(*m*, *k*) represents the value of the *k*-th frequency point in the *m*-th time slice of the short-time fractional Fourier transform domain, *X*(*m*,:) represents all frequency point values in the *m*-th time slice of the short-time fractional Fourier transform domain, and mid(·) represents the median (i.e., the median point is taken after the data are arranged in descending or ascending order).

Considering that the interference signal satisfies the characteristics of narrowband signals in each time slice, the method proposed in reference [25] can be used to identify the interference signal. After interference recognition, the result of interference suppression at *k* frequency points in the *m*-th time slice is

(9) $$\hat{X}(m,k)=X(m,k)W(m,k)$$

After interference suppression, the data are subjected to a short-term fractional Fourier inverse transform to recover useful echo signals.

### 3.3. Interference Suppression Evaluation Criteria

To effectively measure interference suppression algorithms, it is necessary to establish evaluation criteria for interference suppression. Two evaluation indicators are introduced, the Signal Distortion Ratio (SDR) [12,26] and Signal Noise Ratio (SNR) [13,26], and we then provide evaluation criteria for defining interference suppression. The SDR before and after interference suppression is defined as

(10) $$\mathrm{SDR}=10log_{10}\left(\frac{\sum {\left|d_{0}\left(n\right)-\hat{d}\left(n\right)\right|}^{2}}{\sum {\left|d_{0}\left(n\right)\right|}^{2}}\right)$$

where $\hat{d}\left(n\right)$ represents the signal after interference suppression and $d_{0}\left(n\right)$ represents the interference-free signal interference. The SNR after interference suppression is defined as

(11) $$\mathrm{SNR}=10log_{10}\left(\frac{{\left|y\right|}^{2}}{{\left|\hat{y}\right|}^{2}}\right)$$

where *y* represents the amplitude of the strong scattering point signal after distance and azimuth pulse compression and $\hat{y}$ represents the average amplitude of the signal around the strong scattering point. According to the concepts of the SDR and SNR, a good interference suppression method should have a small SDR and a big SNR.

## 4. Results

In this section, the algorithm’s effectiveness is verified through simulation. The *S*(*t*) and *I*(*t*) in Equation (1) are set as follows:

(12) $$\left\{\begin{array}{c} S(t)=\exp\left(j0.80\pi t\right)\exp\left(j0.0002\pi t^{2}\right)\\ I(t)=2\exp\left(j0.76\pi t\right)\exp\left(j0.0002\pi t^{2}\right) \end{array}\right.$$

**Figure 2:**
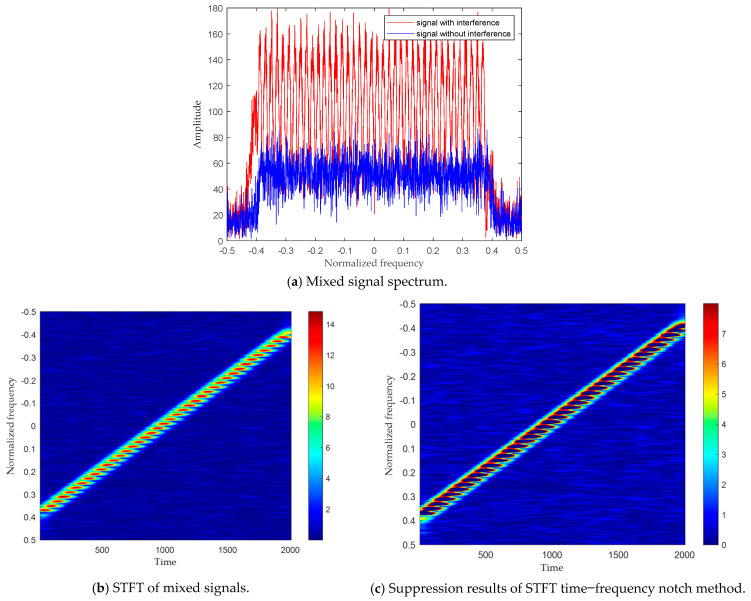

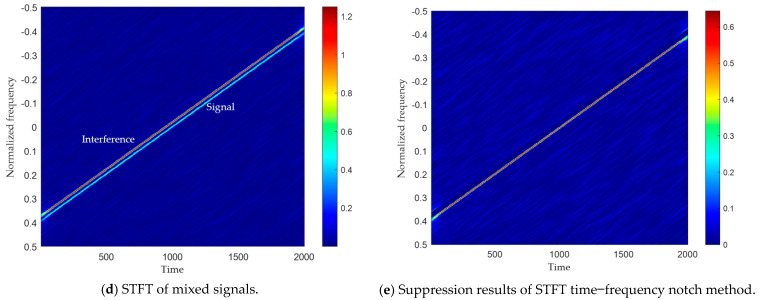
Comparison of two algorithms.

The signal *S*(*t*) and the interference *I*(*t*) have the same frequency modulation rate, are parallel in the time–frequency plane, and have minimal spacing. Figure 2 compares the interference suppression effects of the proposed algorithm and the STFT algorithm. Figure 2a shows the spectrum of both signals with and without interference. As shown in (a), the amplitude of the polluted signal is much greater than the signal without interference. Traditional one-dimensional signal processing methods are difficult to distinguish from signal interference. Figure 2b–e use different mathematical theories to transform signals into time–frequency domains; (b) and (c) utilize STFT to transform signals into the TF plane and show interference suppression methods using notch filtering. There is overlap between the interference and the signal in the time–frequency plane, resulting in the loss of useful echo signals while suppressing interference. Figure 2d,e show the results in the TF plane by STFrFT. Due to the short time window, the resolution of the signal in the time–frequency plane is improved without the influence of cross terms, making the support area of the interference and the useful signal distinguishable. Therefore, while suppressing the interference, the signal is protected by the adaptive gain coefficient, W.

The SNR and SDR for Figure 2 are listed in Table 1. It is observed that the proposed algorithm has a greater SNR and lower SDR. Specifically, the proposed algorithm has a 12.07 dB improvement on the STFT interference suppression algorithm. In addition, the proposed algorithm suffers a 17.98 dB lower SDR against STFT. It can be concluded that the proposed algorithm outperforms the STFT algorithm.

FrFT is a generalized form of traditional Fourier transform (FT) with a real fraction order, *α*. Therefore, FrFT inherits the FT’s computational burden, *O*(*N* log *N*), where *N* is the sequence length. Similarly, the computational complexity of STFrFT depends on its corresponding STFT operations, *O*(*N* log *N*). Since the computational cost of the short time window is *O*(*N*), the computational complexity of this STFrFT is *O*(*N*^2^ log *N*). That is, STFrFT has the exact same computational cost as STFT.

## 5. Analysis of Measured Data

**Figure 3:**
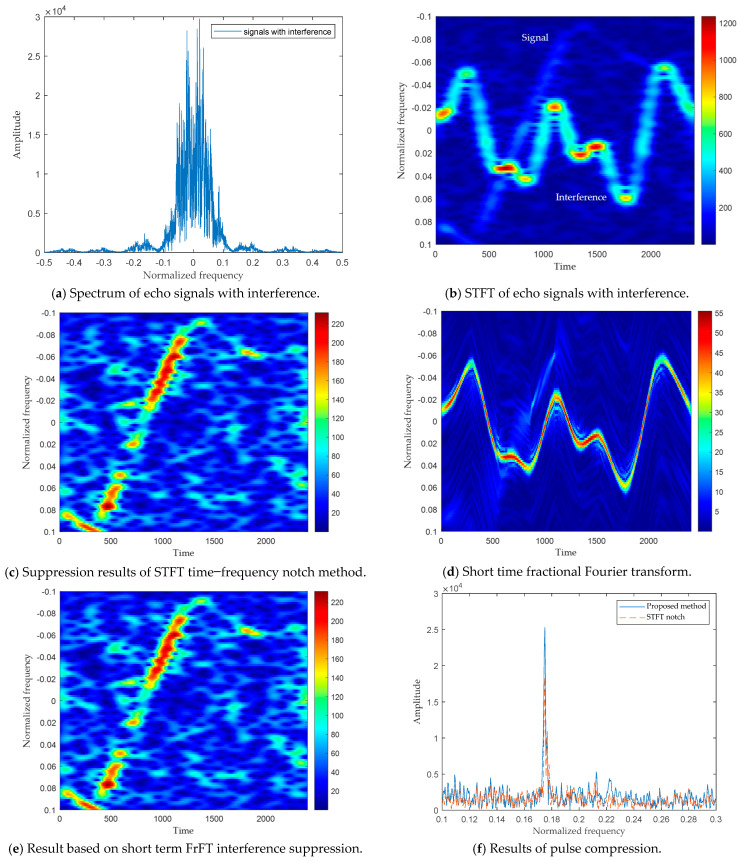
Comparison of STFT and short time FrFT.

In this section, measured data is used to verify the performance of the proposed method. The raw data are a 19-by-2048 matrix. Figure 3a shows the spectrum of the echo signal, which indicates that the signal is submerged in interference and cannot be distinguished. Figure 3b,c show the STFT notch filtering method that transforms the signal into the time–frequency plane. The interference and the useful signal only overlap for a short period of time. However, due to the low time–frequency resolution of STFT, a large part of the useful signal that overlaps with the interference signal is also filtered out while suppressing interference. Figure 3d,e show the proposed algorithm. Compared with Figure 3b, the time–frequency resolution of the interference signal has been significantly improved, resulting in only a tiny loss of useful signal energy that overlaps with the interference when suppressing the interference signal. Figure 3f shows the results of pulse compression using two algorithms. Due to the slight loss of signal energy in the proposed algorithm, the energy of the pulse-compressed signal is greater than that of the STFT time–frequency notch filtering method. 

Due to the inability to obtain raw measured interference-free data, Table 2 only examines the SNR of the proposed algorithm. From Table 2, it can be obtained that the SNR of the proposed algorithm is 1.29 dB greater than that of the STFT time–frequency notch filtering method. Therefore, the proposed method can effectively retain useful echo data while suppressing interference.

## 6. Conclusions

This article proposes an interference suppression algorithm based on short-term fractional Fourier transform and adaptive gain control. The algorithm avoids complex parameter modeling by transforming the echo signal into the short-time fractional Fourier transform domain, achieving the detection and suppression of interference signals within the transform domain. Finally, short-time fractional Fourier inverse transform is used to obtain the echo signal after interference suppression. The simulation and measured data processing results show that the interference suppression method proposed in this paper performs better than the STFT time–frequency notch filtering method while effectively retaining useful signals.

## Figures and Tables

**Table 1:** Performance comparison between the two algorithms.

Evaluation Criteria	STFT Interference Suppression Method	Proposed Algorithm
SNR (dB)	30.17	42.24
SDR (dB)	−0.44	−18.42

**Table 2:** Comparison of two algorithms.

Evaluation Criterion	STFT Interference Suppression Method	Proposed Algorithm
SNR (dB)	22.72	24.01

## Data Availability

The data presented in this study are available on request from the corresponding author. The data are not publicly available due to privacy.
